# Clustering of cardio-metabolic risk factors and pre-diabetes among U.S. adolescents

**DOI:** 10.1038/s41598-021-84128-6

**Published:** 2021-03-03

**Authors:** Chibo Liu, Susu Wu, Xiao Pan

**Affiliations:** Department of Clinical Laboratory Medicine, Taizhou Municipal Hospital, Taizhou, China

**Keywords:** Endocrinology, Risk factors

## Abstract

Few studies have assessed the association between clustering of cardio-metabolic risk factors (CMRFs) and pre-diabetes in children or adolescents. We aimed to examine the association between clustering of CMRFs and pre-diabetes among U.S. adolescents. Data were available for 5,633 U.S. adolescents aged 12–19 years from the National Health and Nutrition Examination Surveys 1999–2014. Pre-diabetes was defined as impaired fasting glucose (IFG) (fasting plasma glucose 100–125 mg/dL), impaired glucose tolerance (IGT) (2-h plasma glucose 140–199 mg/dL) or elevated hemoglobin A1c (HbA1c) (HbA1c 5.7–6.4%). The individual CMRFs considered in the present study were as follows: waist-to-height ratio, blood pressure, triglycerides, and high-density lipoprotein cholesterol. CMRFs were defined based on the modified National Cholesterol Education Program (NCEP) criteria or the modified International Diabetes Federation (IDF) criteria. Logistic regression analysis was used to examine the association between clustering of CMRFs and pre-diabetes with adjustment for potential covariates. Among 5633 adolescents, 11.4% had IFG, 4.7% had IGT, 4.5% had elevated HbA1c and 16.1% had pre-diabetes. Compared with adolescents with no CMRFs, the odds ratios (ORs) with 95% confidence intervals (CIs) for pre-diabetes across the clustering of CMRFs (i.e., 1, 2, 3, and 4) were 1.32 (1.03–1.68), 2.07 (1.55–2.76), 2.52 (1.69–3.76), and 5.41 (3.14–9.32), respectively, based on the modified NCEP criteria. The corresponding ORs with 95% CIs were 1.16 (0.89–1.51), 1.78 (1.35–2.36), 3.07 (1.89–4.98) and 12.20 (3.93–37.89), respectively, based on the modified IDF criteria. The present study suggests that the clustering of CMRFs is associated with increased pre-diabetes among U.S. adolescents. It might be necessary for effective strategies and measures targeting adolescents with clustering of CMRFs, including those with less than 3 risk factors.

## Introduction

Diabetes has been a serious public health issue among the U.S. population. The economic burden associated with diabetes-related problems in the U.S. exceeded 322 billion dollars in 2012^1^. In addition, according to the Centers for Disease Control and Prevention in the U.S., there were approximately 84.1 million adults with pre-diabetes in 2015^2^. The prevalence of pre-diabetes among U.S. adolescents increased significantly from 1999 to 2014 (1.9% to 5.0%)^3^. Fortunately, with early intervention, pre-diabetes can be reversible, especially in children and adolescents^4^. Thus, it is important for early intervention to build healthy habits that might prevent them from having diabetes and diabetes-related chronic diseases in adulthood.

With the epidemic of pediatric obesity, multiple cardio-metabolic risk factors (CMRFs) have been clustered in children and adolescents. Metabolic syndrome (MetS) is diagnosed when three or more CMRFs appear. However, the clinical utility of MetS in the pediatric population has been questioned^5^. Major concerns include a lack of uniformed criteria with many different pediatric MetS definitions, the instability of the dichotomous diagnosis, and no uniformed treatment for MetS rather than weight loss. Furthermore, although MetS in adults has been shown to be a well predictor for development of type 2 diabetes mellitus^6^, its values in children and adolescents are still challenging. Previous studies reported that MetS had low sensitivity in identifying adolescents with pre-diabetes^7^. Of note, the Bogalusa Heart Study showed significant increase in severity of atherosclerotic lesions associated with the increased clustering of CMRFs^8^. Thus, the American Academy of Pediatrics in 2017 recommends that it is better to focus on the concept of clustering of CMRFs rather than the definition of MetS in children or adolescents^9^. Identifying children or adolescents with multiple CMRFs may help target focused interventions on those with the highest risk for cardio-metabolic disease^9^. However, it is still unclear whether the risk of pre-diabetes in adolescents also increases with the clustering of CMRFs.

Therefore, in the present study, we aimed to assess the association between clustering of CMRFs and risk of pre-diabetes among adolescents using data from the National Health and Nutrition Examination Surveys (NHANES) 1999–2014.

## Methods and materials

### Study population

The NHANES is a continuous, nationwide survey conducted by the National Center for Health Statistics (NCHS) of the Centers for Disease Control and Prevention. A stratified, multistage probability sampling design was used to obtain a representative sample of the civilian non-institutionalized resident population in the U.S. It has been conducted in 2-year cycles since 1999 with the goal of monitoring the health and nutritional status of U.S. children, adolescents, and adults. Participants were invited to complete household interviews, followed by physical examinations and laboratory tests. More detailed information about the NHANES is available online^10^. Written informed consent was obtained from adolescents aged 18 years or older. For adolescents aged 12 to 17 years, the content was signed by both adolescents and their parents/guardians. The survey protocol was approved by the NCHS Research Ethics Review Board. The NHANES data are publicly available without personal identifiable information and exempt under the ethical board review of the corresponding author’s institution. All methods were carried out in accordance with relevant guidelines and regulations.

This study was limited to adolescents aged 12–19 years. To produce reliable estimates, we combined the 1999–2014 NHANES data for analysis^11^. We used multiple imputation method to fill in data if participants with missing information on all variables of interests. After the exclusion of participants with diagnosed (fasting plasma glucose (FPG) ≥ 126 mg/dL, or 2-h glucose ≥ 200 mg/dL, or Hemoglobin A1c (HbA1c) ≥ 6.5%)^12^ and undiagnosed diabetes (self-reported physician-diagnosis of diabetes) from the analysis, a total of 5,633 adolescents with normal glucose or pre-diabetes were finally included in this study.

### Measurement of CMRFs

CMRFs considered in the present study were as follows: waist-to-height ratio (WHtR), blood pressure (BP), triglycerides (TG), and high-density lipoprotein cholesterol (HDL-C). The physical examinations and laboratory tests were conducted at a mobile examination center. Waist circumference (WC) was measured to the nearest 0.1 cm using a standard tape. The tape was extended around the waist at the level of the uppermost lateral border of the ilium, and the reading was recorded at minimal respiration^13^. WHtR was calculated as WC divided by height. BP was measured by physicians using a mercury sphygmomanometer with the participants sitting straight and arm at the level of heart after at least 5 min rest. The average of the last two readings was used when three measures were taken^13^. Details of blood collecting and processing in NHANES are available elsewhere^13^. In brief, blood sample was drawn by trained nurses from adolescents who had completed at least an 8-h fast. Blood specimens were placed in − 70 °C Freezer until testing. TG and HDL-C were measured enzymatically.

### Measurement of glucose and hemoglobin A1c

FPG and HbA1c were measured using the blood sample drawn in the morning after at least an 8-h fast. Then, an oral glucose tolerance test was administered. Participants were asked to drink 75 g glucose and two hours later, a second blood sample was drawn to obtain 2-h PG. FPG and 2-h PG were measured using hexokinase enzymatic methods and HbA1c was measured using high-performance liquid chromatography methods^13^. As glucose measurement methods have changed over time, to ensure comparability with earlier NHANES, we calibrated glucose data using regression equations provided in the NHANES data documentation^14,15^.

### Potential confounders

Covariates adjusted in this study include sex, age, race/ethnicity (Hispanic, Non-Hispanic white, Non-Hispanic black, and others), and survey year, which were collected using the questionnaire.

### Definitions of pre-diabetes

Impaired fasting glucose (IFG) was defined as having FPG 100–125 mg/dL^12^. Impaired glucose tolerance (IGT) was defined as having 2-h PG 140–199 mg/dL^12^. Elevated HbA1c was defined as having HbA1c 5.7–6.4%^12^. According to the American Diabetes Association (ADA) criteria, individuals with IFG, IGT, or elevated HbA1c and without diagnosed or undiagnosed diabetes were classified as having pre-diabetes^12^.

### Definitions of CMRFs

Since there is no universal definition for CMRFs in children and adolescents, we used two international MetS criteria to determine whether participants had any of the four CMRFs (i.e. central obesity, elevated TG, low HDL-C, and elevated BP) that are components of the MetS. The two international MetS criteria included the modified International Diabetes Federation (IDF) criteria^16^ and the modified National Cholesterol Education Program (NCEP) criteria^17^. For the modified IDF definition, central obesity was defined as WHtR ≥ 0.50; elevated BP was defined as systolic BP ≥ 130 mmHg or diastolic BP ≥ 85 mmHg; elevated TG was defined as TG ≥ 150 mg/dL; and low HDL-C was defined as HDL-C < 40 mg/dL for those aged < 16 years or HDL-C < 40 mg/dL in males and < 50 mg/dL in females for those aged ≥ 16 years^16^. For the modified NCEP definition, central obesity was defined as WHtR ≥ 0.50; elevated BP was defined as systolic/diastolic BP ≥ 90^th^ percentile; elevated TG was defined as TG ≥ 110 mg/dL; and low HDL-C was defined as HDL-C ≤ 40 mg/dL^17^.

The clustering of CMRFs was defined as the sum of the four individual risk factors with each individual risk factor categorized as 0 vs. 1. Thus, five categories were created, ranging from 0 to 4.

### Statistical analysis

Continuous variables were presented as means (standard errors [SE]), and the categorical variables were expressed as percentages. Differences in FPG, 2-h PG, and HbA1c across the five categories of CMRFs were compared using covariance analysis adjusted for sex, age, race/ethnicity, and survey years. Chi-square test was used for comparison of categorical variables across the five categories of CMRFs. In addition, logistic regression models were used to estimate the odds ratios (ORs) with 95% confidence intervals (CIs) of clustering of CMRFs associated with IFG, IGT, elevated HbA1c, and pre-diabetes, respectively, with adjustment for potential confounding factors. The NHANES uses a multistage sampling design, thus the sample design variables (strata, cluster and weights) were accounted for in the analyses for generalizability of the estimates. We performed all analyses using SAS version 9.3 (SAS, Cary, North Carolina, USA). Two-sided *P* value < 0.05 was considered to be statistically significant.

## Results

Of the 5,633 adolescents included in this study, 11.4% had IFG, 4.7% had IGT, 4.5% had elevated HbA1c, 13.0% had both IFG and IGT, 14.8% had both IFG and elevated HbA1c, 6.2% had both IGT and elevated HbA1c, and 16.1% had pre-diabetes (either IFG, IGT, or elevated HbA1c). Table 1 shows the characteristics of U.S. adolescents aged 12–19 years according to the clustering of CMRFs (based on NCEP criteria). Significant differences in all characteristics were found across the five categories of CMRFs (all *P* < 0.0001). In general, participants with clustering of CMRFs were more likely to be male, adolescents aged 16–19 years, Non-Hispanic white, to have abnormal anthropometric indices (BMI, WC, BP) and lipid profiles (TG and HDL-C) (all *P* < 0.0001).

**Table 1:** Characteristics of U.S. adolescents aged 12–19 years, NHANES 1999–2014.

	Clustering of cardio-metabolic risk factors					*P*-value
	0	1	2	3	4	
*N*	2540	1765	831	401	96	
**Sex, %**						< 0.0001
Male	50.7	46.1	53.5	63.6	86.5	
Female	49.3	53.9	46.5	36.4	13.5	
**Age (years), %**						< 0.0001
12–15	57.0	44.6	42.2	37.5	32.1	
16–19	43.0	55.4	57.8	62.5	67.9	
**Race/ethnicity, %**						< 0.0001
Hispanic	15.5	18.7	23.7	23.0	17.6	
Non-Hispanic white	59.7	60.5	57.2	62.9	70.6	
Non-Hispanic black	15.9	15.2	13.4	8.9	5.6	
Others	8.9	5.6	5.6	5.2	6.2	
BMI, kg/m^2^	20.2 (0.1)	24.6 (0.2)	27.1 (0.3)	30.7 (0.5)	33.0 (0.6)	< 0.0001
WC, cm	72.5 (0.2)	84.0 (0.4)	90.7 (0.7)	101.0 (1.1)	107.9 (1.6)	< 0.0001
WHtR	0.438 (0.001)	0.505 (0.003)	0.544 (0.004)	0.599 (0.006)	0.616 (0.010)	< 0.0001
SBP, mmHg	105.3 (0.3)	110.0 (0.3)	113.0 (0.6)	117.0 (0.7)	126.5 (0.7)	< 0.0001
DBP, mmHg	60.5 (0.3)	61.1 (0.4)	61.9 (0.5)	63.4 (0.8)	64.0 (1.5)	< 0.0001
TG, mg/dL	63.3 (0.7)	82.4 (1.4)	117.3 (3.2)	157.4 (5.6)	182.1 (8.7)	< 0.0001
HDL-C, mg/dL	56.9 (0.3)	51.1 (0.4)	44.4 (0.5)	38.3 (0.5)	33.7 (0.6)	< 0.0001

Continuous variables are expressed as mean (SE).

**Figure 1:**
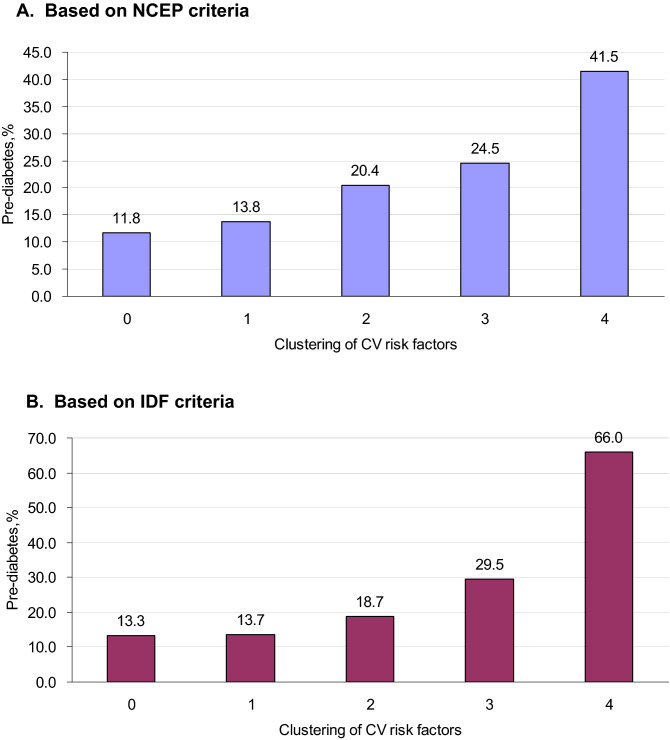
Prevalence of pre-diabetes according to clustering of cardio-metabolic risk factors based on (**A**) NCEP criteria and (**B**) IDF criteria.

Table 2 shows mean FPG, 2-h PG, and HbA1c levels according to the clustering of CMRFs. With the clustering of CMRFs, FPG, 2-h PG and HbA1c levels increased gradually, from 90.7 mg/dL to 95.6 mg/dL, from 92.6 mg/dL to 110.0 mg/dL and from 5.12% to 5.20%, respectively, based on the modified NCEP criteria; the prevalence of pre-diabetes also increased gradually with the clustering of CMRFs. For adolescents with 0, 1, 2, 3 and 4 CMRFs (defined according to the modified NCEP criteria), the prevalence of pre-diabetes was 11.8%, 13.8%, 20.4%, 24.5%, 41.5%, respectively (*P* for trend < 0.001, Fig. 1A). Similar results were found according to the modified IDF criteria (Fig. 1B).

**Table 2:** Mean FPG, 2-h PG and HbA_1C_ levels according to clustering of cardio-metabolic risk factors.

	Clustering of cardio-metabolic risk factors					*P-*value
	0	1	2	3	4	
**NCEP criteria**						
FPG, mg/dL	90.7 (0.2)	91.3 (0.2)	92.1 (0.3)	93.0 (0.5)	95.6 (0.8)	< 0.0001
2-h PG, mg/dL	92.6 (1.5)	96.9 (1.3)	102.2 (1.7)	111.8 (3.1)	110.0 (5.3)	< 0.0001
HbA_1C_, mg/dL	5.12 (0.01)	5.14 (0.01)	5.15 (0.01)	5.19 (0.02)	5.20 (0.05)	0.0622
**IDF criteria**						
FPG, mg/dL	90.9 (0.2)	91.4 (0.2)	92.2 (0.3)	93.3 (0.7)	98.1 (1.1)	< 0.0001
2-h PG, mg/dL	93.6 (1.4)	98.5 (1.2)	103.2 (2.0)	114.8 (4.2)	106.0 (13.9)	< 0.0001
HbA_1C_, mg/dL	5.13 (0.01)	5.14 (0.01)	5.17 (0.02)	5.19 (0.04)	5.29 (0.06)	0.0220

Adjusted for sex, age, race/ethnicity, and survey years.

Logistic regression analyses adjusted for sex, age, race/ethnicity, and survey years showed that the risk of pre-diabetes tended to increase with the clustering of CMRFs (Table 3). Compared with healthy adolescents with no CMRFs, the ORs (95% CIs) for pre-diabetes across the clustering of CMRFs (i.e. 1, 2, 3, and 4) were 1.32 (1.03–1.68), 2.07 (1.55–2.76), 2.52 (1.69–3.76), and 5.41 (3.14–9.32), respectively, based on the modified NCEP criteria. The corresponding values were 1.16 (0.89–1.51), 1.78 (1.35–2.36), 3.07 (1.89–4.98) and 12.20 (3.93–37.89), respectively, based on the modified IDF criteria (the limited sample size of 18 in the fifth category of CMRFs resulting in the wider 95%CI in this category). Similar results were observed for IFG, IGT and elevated HbA1c.

**Table 3:** Odds ratios (95% CI) of pre-diabetes according to clustering of cardio-metabolic risk factors.

	Clustering of cardio-metabolic risk factors				
	0	1	2	3	4
**NCEP criteria**					
IFG	1.00 (ref)	1.35 (1.02–1.78)	1.77 (1.25–2.52)	1.97 (1.28–3.04)	5.22 (2.93–9.30)
IGT	1.00 (ref)	0.95 (0.48–1.91)	2.14 (0.87–5.25)	4.87 (2.06–11.53)	7.61 (2.20–26.38)
Elevated HbA_1C_	1.00 (ref)	1.61 (1.07–2.43)	2.76 (1.71–4.44)	3.41 (1.85–6.26)	4.67 (1.77–12.29)
Pre-diabetes	1.00 (ref)	1.32 (1.03–1.68)	2.07 (1.55–2.76)	2.52 (1.69–3.76)	5.41 (3.14–9.32)
**IDF criteria**					
IFG	1.00 (ref)	1.20 (0.88–1.64)	1.61 (1.14–2.29)	2.28 (1.38–3.79)	12.83 (4.59–35.82)
IGT	1.00 (ref)	1.17 (0.59–2.32)	1.61 (0.65–3.94)	5.67 (2.11–15.23)	7.32 (1.28–41.99)
Elevated HbA_1C_	1.00 (ref)	1.41 (0.98–2.04)	2.74 (1.77–4.24)	3.32 (1.57–7.02)	6.15 (1.35–28.11)
Pre-diabetes	1.00 (ref)	1.16 (0.89–1.51)	1.78 (1.35–2.36)	3.07 (1.89–4.98)	12.20 (3.93–37.89)

Adjusted for sex, age, race/ethnicity, and survey years.

## Discussion

In this pooled analysis of 8 nationally representative population samples of the U.S. adolescents, we found that the risk of pre-diabetes tended to increase with the clustering of CMRFs. Our findings emphasize the need for effective strategies and measures targeting adolescents with clustering of CMRFs to reduce risk of pre-diabetes.

There has been controversy on determining the optimal method to define pre-diabetes in adolescents. IFG, IGT, and elevated HbA1c have been proposed to have distinct etiological mechanisms^18^ and have poor agreement as indicators of pre-diabetes^3,19,20^. A study using data from NHANES 1999–2014 showed different temporal trends of pre-diabetes as defined by IFG or elevated HbA1c^3^. Although HbA1c has the advantages of convenience and less variability during illness, it also has the limitations of lower sensitivity and greater cost, which may affect the number of adolescents classified as patients^12^. Thus, in the present study we investigated the relationship between clustering of CMRFs and risk of pre-diabetes using IFG, IGT and elevated HbA1c, separately, as well as the combination. The results showed that all the indicators were strongly associated with clustering of CMRFs. The assessment of clustering of CMRFs in adolescents may aid pediatricians to identify and treat those with potential risk of pre-diabetes.

We also found that the relationship was not limited to three or more CMRFs. Instead, compared with adolescents with no CMRF, those with two CMRFs also had a significantly higher risk of pre-diabetes. A previous study conducted using NHANES data 2005–2006 (n = 777) also demonstrated that adolescents with two or more of the four CMRFs had a higher prevalence of pre-diabetes than those with no CMRF^21^. Several other studies used subclinical markers of cardiovascular disease as the outcome and showed similar results. A study conducted among 474 adolescents and found that participants who had two or more CMRFs had greater vascular stiffness and wall thickness^22^. Another study using data from the Bogalusa Heart Study (n = 204) showed that the atherosclerotic process was accelerated in an exponential manner with the increasing number of CMRFs^8^. Therefore, it is important to note that the risk among adolescents with less than 3 risk factors may be overlooked when using the traditional dichotomous MetS definition, which was referred to the presence of three or more risk factors. A study performed among 461 overweight adolescents aged 10–18 years showed that the best model for diagnosing increased intima-media thickness was the sum of the components of MetS, while the dichotomized variable MetS reduced the diagnostic accuracy^23^. Overall, all these findings suggest that in clinical practice, clinicians should focus attention on children and adolescents with CMRFs clustering instead of a dichotomous definition of MetS. Of note, the greatest increase in the risk of pre-diabetes in this study was seen in adolescents with 4 CMRFs, indicating CMRFs clustering may produce a synergistic effect on pre-diabetes, rather than a simple additive effect^24^.

Our study has several strengths. A major strength is the large, population-based sample size obtained by combining 1999–2014 NHANES data. The large sample size allows us to investigate the patterns of the association across gradients of CMRFs. However, several limitations of our study also warrant consideration. First, the cross-sectional nature of NHANES data precluded the causal inference of CMRFs clustering and adolescent pre-diabetes. Further longitudinal studies are needed to clarify the observed association. Second, the single measurement of PFG, 2-h PG and HbA1c may result in misclassification of pre-diabetes, since glucose measures are subject to variability. However, the ADA does not require a repeat measurement to determine pre-diabetes^25^. Third, there were relatively few cases had 4 CMRFs. However, the results were stable when using different criteria in the study. Fourth, we only included 4 CMRFs in our analysis. Further studies should include other risk factors such as lifestyle factors. Fifth, we treated the four risk factors to have the equal weight in determining pre-diabetes, in accord with the definition of MetS in children. Further studies should validate this assumption. Sixth, the statistical significance is set at *P* < 0.05 without consideration of correction for multiple comparisons, which may lead to false positive results.

In conclusion, this study confirms a positive association between the clustering of CMRFs and pre-diabetes among U.S. adolescents. It might be necessary for effective strategies and measures aiming at adolescents with clustering of CMRF, including those with less than 3 risk factors.
